# Dual Substrate Binding stabilizes the Catalytic State in Ketohexokinase

**DOI:** 10.1063/4.0000798

**Published:** 2025-10-27

**Authors:** So Young Bae, Karen N. Allen, Dean R. Tolan

**Affiliations:** Boston University, Boston, MA, USA

## Abstract

Understanding conformational changes in enzymes and associated changes in the active site affecting catalysis has been a long-standing problem in enzymology. One major factor for initiating these conformational changes for many enzymes is substrate binding. Ketohexokinase (KHK) catalyzes the first reaction in fructose metabolism, converting β-D-fructofuranose into fructose 1-phosphate. Recently, the over- ingestion of fructose has been reported as a root cause of a variety of metabolic diseases including Type-2 diabetes, non-alcoholic fatty liver disease, and metabolic syndrome, the consequences of which can be traced back to the KHK-catalyzed reaction. KHK is a homodimer that exists as two isoforms, KHK-A and KHK-C. The C isozyme is mainly expressed in liver and kidney and is the major target for drug development to prevent fructose metabolism. The A isozyme, expressed ubiquitously, has been shown to function as a protein kinase as well as a sugar kinase, albeit with less fructokinase efficiency than KHK-C. The two subunits are connected by the flexible β-clasp domain, whose mobility has been demonstrated to be essential for catalysis. To investigate the mechanism of KHK-A, which had been previously reported to operate without this movement, a structure of mouse KHK-A was determined and the unliganded structure showed that KHK-A undergoes the same conformational changes in the β-clasp domain as KHK-C. Moreover, comparison of this structure to other structures of unliganded KHK enzymes showed a variety of conformations, which indicated that the unliganded enzyme has a fairly flat energy landscape for interconversion of conformers. The question of whether subsequent binding of one or more substrates led to an induced-fit mechanism was answered using macromolecular X-ray crystallography of the enzyme in complex with one, two, or no ligands (ATP, ADP, and/or fructose). Herein, seven new structures of KHK-A and - C with various substrates are presented. When comparing the conformations of the β-clasp domain among these structures, the structures bound to a single substrate (fructose or ATP) attained conformations similar to those observed for the unliganded structures, and only the ternary structure attained what might be considered the catalytically competent conformation. This is consistent with a model where the amount of intrinsic binding energy needed to achieve the catalytically competent conformation is provided only by the binding of both substrates.

